# Supplementary material for: Neural dynamics of causal inference in the macaque frontoparietal circuit
Source: eLife. 2022 Oct 24;11:e76145. doi: 10.7554/eLife.76145 (PMC9642994; doi:10.7554/eLife.76145)
Supplement: Supplementary file 1. [file elife-76145-supp1.docx]

**Supplementary file 1**

**Supplementary file 1-Table 1. Model parameters and fitting evaluations of four models for monkeys.**

| subject | Causal inference (model averaging) | | | | | | Forced fusion | | | | |
| --- | --- | --- | --- | --- | --- | --- | --- | --- | --- | --- | --- |
|  | relBIC_group_ | EP | R^2^ | 𝝈_P_ | 𝝈_V_ | *Prior* | relBIC_group_ | EP | R^2^ | 𝝈_P_ | 𝝈_V_ |
| Monkey H | 0 | 1 | 0.96±0.0017 | 7.72±0.14 | 5.83±0.090 | 0.999±0.0004 | 329.52 | 0 | 0.93±0.0039 | 9.87±0.24 | 9.02±0.23 |
| Monkey N | 0 | 1 | 0.93±0.0080 | 9.56±0.16 | 4.93±0.15 | 0.86±0.026 | 812.34 | 0 | 0.37±0.36 | 11.34±0.10 | 10.46±0.13 |
| Monkey S | 0 | 1 | 0.96±0.0022 | 8.98±0.14 | 5.72±0.22 | 0.98±0.012 | 290.04 | 0 | 0.94±0.0027 | 10.10±0.14 | 8.34±0.17 |
| subject | Full segregation (proprioceptive only) | | | | Full segregation (visual only) | | | |  |  |  |
|  | relBIC_group_ | EP | R^2^ | 𝝈_P_ | relBIC_group_ | EP | R^2^ | 𝝈_V_ |  |  |  |
| Monkey H | 1876.10 | 0 | 0.44±0.026 | 14.43±0.15 | 1677.04 | 0 | 0.58±0.019 | 14.51±0.091 |  |  |  |
| Monkey N | 1902.82 | 0 | 0.53±0.018 | 14.76±0.070 | 1739.40 | 0 | 0.51±0.078 | 14.38±0.095 |  |  |  |
| Monkey S | 1937.32 | 0 | 0.39±0.040 | 14.72±0.066 | 1440.47 | 0 | 0.71±0.016 | 12.75±0.28 |  |  |  |

The model parameters and *R*^2^ were averaged across days for monkeys; data are presented as the means ± the standard errors of the means. The relBICgroup was the summation of all days’ BIC for monkeys.

Abbreviations: 𝝈_P_, the standard deviation of the proprioception likelihood; 𝝈_V_, the standard deviation of the vision likelihood; *P_prior_*, the prior probability of a common source; relBICgroup, Bayesian information criterion at the group level; EP, exceedance probability; *R*^2^, coefficient of determination.
